# Persistent viremia in an immunocompetent patient with inherited chromosomally integrated HHV-6B

**DOI:** 10.1099/acmi.0.000256

**Published:** 2021-08-04

**Authors:** Michele Obeid, Inderdeep Gakhal, Philip J. McDonald

**Affiliations:** ^1^​ Department of Internal Medicine, Hurley Medical Center/Michigan State University, Flint, Michigan, USA

**Keywords:** chromosomally integrated, Human herpesvirus, HHV-6, viremia

## Abstract

Human herpesvirus-6 (HHV-6), the virus which causes roseola, has traditionally been associated with benign and self-limited childhood illness. However, HHV-6 establishes lifelong latency and can reactivate in immunocompromised adult patients. In about 1% of cases, it integrates into the human genome as inherited chromosomally integrated HHV-6 (iciHHV-6). We report the case of a 70-year-old man presenting with altered mental status and agitation. His infectious workup revealed a cerebrospinal fluid sample positive for HHV-6 with virus detectable in the blood as well. He was subsequently treated with ganciclovir. HHV-6 viremia (DNAemia) persisted, and the antiviral medications were switched to foscarnet under the assumption of treatment failure due to drug resistance. After several admissions to the hospital for the same complaint, and after noticing that DNAemia persisted despite adequate treatment for HHV-6, infectious disease specialists ordered testing for chromosomally integrated virus. Test results confirmed the presence of iciHHV-6, explaining his consistently elevated serum viral load. Primary HHV-6 infection in adults causes a transient increase in viral load with resolution and clearance after a few weeks while iciHHV-6 is characterized by persistent detection of viral DNA at a high copy number. Individuals with iciHHV-6 can develop HHV-6 disease and are at increased risk for active viral replication when treated with immunosuppressive medications, but only mRNA testing, which is not widely available can differentiate between latent and active infection. This makes the decision to treat challenging in this patient population. When faced with a positive HHV-6 DNA result in the setting of equivocal symptoms, clinicians should consider the possibility of chromosomally integrated virus rather than drug-resistant virus in order to reduce exposure to potentially toxic antiviral medications.

## Introduction

Human herpesvirus-6 (HHV-6) is a double-stranded DNA virus, part of the Herpesviridae family. It is known to be the cause of roseola infantum, a self-limited disease in childhood. This virus establishes latency and can reactivate later in life or in immunosuppressed patients, with potentially severe manifestations such as meningoencephalitis [1]. We report the case of a 70-year-old patient presenting with confusion and agitation, who was found to have inherited chromosomally integrated HHV-6 (iciHHV-6) causing persistent DNAemia. In patients without risk factors for HHV-6 disease, it is important to consider the possibility of chromosomally integrated virus rather than drug-resistant virus when deciding to treat for HHV-6 encephalitis in order to reduce exposure to potentially toxic antiviral medications. In individuals with iciHHV-6, the diagnosis of HHV-6 encephalitis and the decision to treat are based on clinical judgement if the mRNA assay needed to evaluate for active disease is not readily available.

## Case presentation

This is the case of a 70-year-old man who presented to the emergency department with sudden confusion and agitation of 1 day duration. In the initial assessment, vital signs were significant for fever (38.8 C). Physical examination revealed a drowsy elderly patient who was easily arousable and oriented to person. He was able to move all extremities against gravity and no nuchal rigidity was noted. His reflexes were +2 throughout and Babinski sign was negative bilaterally. No rashes or skin lesions were appreciated. Suddenly, while under evaluation in the emergency department, the patient had a generalized tonic-clonic seizure lasting a few minutes.

Initial blood work showed a normal complete blood count (CBC) with differential counts, and an unremarkable comprehensive metabolic panel including globulin levels. Urinalysis was negative for pyuria and bacteriuria. Urine drug screen was also negative. His HIV test was negative. Computed tomography (CT) scan of the head was negative for any acute process. CT angiography of the head and neck was negative for stenosis, occlusion, aneurysm, arteriovenous malformation or dissection. Magnetic resonance imaging (MRI) of the brain showed advanced cerebral atrophy with extensive chronic microvascular ischaemic changes, no evidence of haemorrhage and nonspecific micro-haemorrhages mainly over the deep structure consistent with the sequelae of traumatic brain injury (TBI).

Blood cultures were drawn and the patient underwent a lumbar puncture. He was then started on empiric antibiotics (vancomycin, ampicillin and ceftriaxone) in addition to acyclovir. Cerebrospinal fluid (CSF) profile showed elevated glucose of 161 mg dl^−1^ (normal range 40–70 mg dl^−1^), elevated protein of 71 mg dl^−1^ (normal range 15–45 mg dl^−1^), WBC count of 2 (normal range <10 per cm^3^) with 63% lymphocytes. The CSF sample was positive for HHV-6 on the BioFire FilmArray Meningitis/Encephalitis (ME) Panel (bioMérieux; Marcy-l′Étoile, France). The patient was intubated, transferred to the ICU and was switched to ganciclovir for treatment of suspected HHV-6 encephalitis. Antibiotics were discontinued.

There was no significant improvement in the patient’s mentation despite ganciclovir therapy. Five days later, his HHV-6 quantitative PCR (qPCR) performed on plasma, showed 5.1 log_10_ copies/ml (116 000 copies/ml) of HHV-6B DNA in the plasma and 4.5 log_10_ copies/ml (29 700 copies/ml) in the CSF. A repeat plasma HHV-6 PCR remained elevated at 5.7 log_10_ copies/ml (487 000) copies after 12 days of treatment, raising concern for treatment failure. Although the persistent high viral load could also be explained by iciHHV-6, no HHV-6 mRNA testing was available at the time to determine whether the patient had both ciHHV-6 and an active infection; therefore, the decision was made to continue treatment based on his clinical symptoms and ganciclovir was changed to foscarnet. The patient’s mentation improved to baseline per his family and a repeat HHV-6 plasma PCR showed improvement with 4.6 log_10_ copies/ml (38 000 copies/ml) detected. He received a total of 14 days of foscarnet therapy and was discharged to home.

A few weeks after his discharge, the patient presented again to the emergency room in a postictal state. He was nonadherent to his antiepileptic regimen and had a breakthrough seizure. At that time, the infectious disease specialist did not recommend any further workup for encephalitis. Three months later, the patient was admitted again for altered mental status. According to his family, he was slower to respond and more lethargic than usual. HHV-6 plasma PCR was repeated and showed persistent DNAemia with 4.7 log_10_ copies/ml (54 300 copies/ml) detected. Despite this result and his encephalopathy, the patient was haemodynamically stable without systemic signs of infection. Infectious disease specialists discussed his case and agreed that his multiple admissions for encephalopathy were unlikely secondary to HHV-6 encephalitis given the lack of correlation of viral load or antiviral treatment with his symptoms. Their decision was to send an HHV-6 chromosomal integration assay and hold further treatment pending the result. Meanwhile, the patient’s mentation improved, and he was discharged home. Following discharge, the result of the chromosomal integration assay performed through whole blood qPCR at the University of Washington’s Department of Laboratory Medicine was received; the HHV-6 virus-to-cell ratio was approximately 1:1, confirming our suspicion for iciHHV-6.

## Discussion

HHV-6 was first isolated in 1986 from the serum of six people with various lymphoproliferative disorders. HHV-6B seems to be transmitted through saliva and possibly genital secretions. We now recognize two closely related viral species, HHV-6A and HHV-6B, with HHV‐6B accounting for the majority of documented primary HHV‐6 infections [1–3]. In this paper, these two viruses will be referred to collectively as ‘HHV-6’. HHV‐6A and HHV-6B use the CD46 and CD134 molecules, respectively, as cellular receptor [4]. It infects primarily CD4 +T lymphocytes, as well as monocytes and macrophages. While most herpesviruses achieve latency as an episome in the nucleus; HHV-6 can, in about 1% of the population (0.2–2.9% depending on the region studied), uniquely integrate itself into the chromosome thus creating the potential to be vertically transmitted within the germ-line [5–9].

Infection with HHV-6 frequently occurs during childhood with older siblings serving as the source of transmission [2]. By the age of 2, the majority of children have been exposed to HHV‐6 (1). The classic presentation of primary HHV-6 infection is roseola infantum also known as sixth disease or exanthem subitum, an illness characterized by high-grade fever for 3–5 days with subsequent rash. Roseola has traditionally been considered a benign childhood illness. However, HHV-6B, the causative agent of roseola, is the leading cause of childhood febrile status epilepticus, which leads to increased risk of both hippocampal injury and subsequent temporal lobe epilepsy [10].

In adults, HHV-6 infection is most often seen in immunocompromised hosts such as haematopoietic stem-cell transplant (HSCT) recipients [11]. It can present with neurological manifestations, including convulsions and encephalitis in non-immunocompromised patients, pneumonitis, syncytial giant-cell hepatitis, disseminated disease or graft dysfunction in immunosuppressed patients, and severe maternal-foetal infection [12]. HHV‐6 infection after solid organ transplantation (SOT) occurs either as a primary or secondary infection: as a reactivation of endogenous latent virus or reinfection with donor‐transmitted virus [13, 14]. HHV-6 infection presenting with encephalitis is rare in immunocompetent patients, but is becoming increasingly reported [15–17].

HHV-6A and HHV-6B can establish lifelong latency through integration into the subtelomeric regions of human chromosomes [6]. If this integration occurs in a germ cell, offspring arising from affected gametes (50 %) will have HHV-6 in each of their own cells and this integration is referred to as inherited chromosomally integrated HHV-6 (iciHHV-6). This integration into human chromosome allows vertical transmission, as well as transmission of the integrated virus from the donor to the recipient through haematopoietic stem-cell transplantation [15]. In a study of 254 infants, 43 (approximately 1.7 %) had congenital HHV-6 infection, with 86 % identified as having iciHHV-6 and 14% as having a transplacental infection [16]. The second group is characterized by low viral loads in their cord blood and peripheral blood as well as a lack of HHV-6 DNA in hair follicles; when an infant has chromosomally integrated virus and a high level of HHV-6 DNA in the blood, it is impossible to determine if they also acquired active infection from the mother. iciHHV-6 is present in 0.5–2% of the population worldwide, with the majority carrying iciHHV-6B [17]. Luppi *et al*. first reported three unrelated cases of chromosomally integrated HHV-6 (iciHHV-6) in 1993 [18]. In one Japanese study, HHV‐6A infection was more prevalent in Japan and the frequency of ciHHV‐6A (57 %) was higher than iciHHV‐6B (43 %) [4]. In addition, eight iciHHV‐6A cases and six ciHHV‐6B cases were found in which the viral genome was integrated in chromosome 22q [19–21]. Most iciHHV‐6 cases in the United States and Europe demonstrated integration into chromosome 17, though the virus has also been shown to integrate into 1q, 6q, 7q, 9q, 10q, 11 p, 18 p, 18q, 19q, 22q and Xp [5, 9, 22–24].

Inherited chromosomally integrated HHV-6 is characterized by persistent detection of the HHV-6 genome at high copy number in whole blood, and in other samples including hair follicles and nail clippings [18, 25]. This highlights the importance of distinguishing between iciHHV-6 and actively replicating virus as during primary infection in infants, or virus reactivation in older individuals; HHV-6 mRNA testing can help determine if HHV-6 is actively replicating in an individual with iciHHV-6 [26]. The chromosomally integrated HHV-6 virus can excise from the chromosome and activate under various conditions such as transplantation, inherited immunodeficiencies [19] or pregnancy [27]. It has been shown that iciHHV-6 in donors or recipients of allogeneic HSCT is a risk factor for acute graft versus host disease (GVHD), and recipients are more likely to experience cytomegalovirus (CMV) reactivation [28].

HHV-6 is included in the most commonly used rapid multiplex PCR panels for meningitis/encephalitis [29]. Establishing HHV-6 infection based on detection from CSF can be challenging since individuals with iciHHV-6 have the virus in every cell; a positive CSF HHV-6 result may simply be due to lysed cells and thus not necessarily reflect active HHV-6 infection. A retrospective study including 15 patients between 27 days to 89 years of age with a positive result for HHV-6 on the CSF PCR panels revealed low clinical probability of true HHV-6 encephalitis despite detectable HHV-6 plasma viral load in 7 of the 11 patients tested [30]. Additional testing for iciHHV-6 may be warranted in these individuals.

Quantitative PCR testing on whole blood is used to determine iciHHV-6 status. High viral loads from whole blood (generally, over 5.5 log_10_ copies/ml) may be seen in both cases of iciHHV-6 and acute infection, however low copy number rules out iciHHV-6 [6, 27]. The correlation between HHV-6 DNA load between plasma and whole blood is poor, and the plasma viral load depends on the time from collection to centrifugation, therefore low copy numbers in plasma cannot be used to rule out iciHHV-6 [31]. Qualitative PCR testing of other cellular samples such as hair follicles or fingernail clipping can also be used to determine iciHHV-6 status [32]. The ratio of viral to human genomes is expected to be about 1:1 (6). Again, HHV-6 mRNA is essential to determine if the virus is actively replicating [26]. In the United States, Coppe Laboratories (Waukesha, WI) now offers an HHV-6 RT-PCR mRNA panel on whole blood, bone marrow and cord blood but similar testing may not be available elsewhere. Non-iciHHV-6 patients with acute infection may have high viral loads, however the viremia is transient and improves within a week, as the acute viral phase subsides. This is rare in immunocompetent adults. Non-iciHHV-6 patients with reactivation extremely rarely have viral loads greater than 5.5 log_10_ copies/ml, and these individuals are uniformly acutely ill.

No antivirals are currently licensed for the specific treatment of HHV-6 [32] and there is no evidence to support the use of antiviral prophylaxis since the cases are largely subclinical, transient and self‐limiting. Antiviral treatment is indicated in the setting of active HHV‐6 encephalitis and other tissue‐invasive diseases and is guided by serial monitoring of HHV‐6 viral loads. This becomes increasingly pertinent in certain clinical situations such as transplant patients, where delirium and encephalitis are commonly caused by HHV-6 [33]. Treatment options include ganciclovir and foscarnet [13]. Most cases respond well to ganciclovir; foscarnet can be used to treat ganciclovir-resistant virus [34]. Ganciclovir resistance mutations can in the U69 kinase gene involved in the initial phosphorylation of ganciclovir, which is necessary for its antiviral action. In CMV, prolonged treatment with ganciclovir may result in antiviral drug resistance when viral replication is incompletely suppressed [35, 36]. The rate of emergence of ganciclovir-resistant HHV-6 appears to be lower, even in HSCT recipients treated with ganciclovir [37]. Combined ganciclovir and foscarnet resistance in CMV is very rare but has been reported [38]. Foscarnet resistance in HHV-6 has been induced *in vitro* through cell culture under increasing concentrations of the drug, and is mediated by mutations in the U38 DNA polymerase [39]. To our knowledge, clinical disease due to foscarnet-resistant HHV-6 has not been described.

There were three possibilities with regard to our patient’s clinical presentation: active HHV-6 disease (encephalitis), iciHHV-6 with concomitant HHV-6 encephalitis, and iciHHV-6 without HHV-6 encephalitis. A lack of sustained reduction in his serum HHV-6 viral loads raised concern for ganciclovir resistance, however foscarnet resistance was considered exceedingly unlikely. Our patient was not immunosuppressed, other than potentially by virtue of his age; he was not on any immunosuppressive medications, his total lymphocytes and globulin levels were within normal limits, HIV testing was negative and there was no history to warrant investigation for hypogammaglobulinemia. Of note, he was taking levetiracetam and lacosamide for seizures related to a prior traumatic brain injury. Levetiracetam does not have intrinsic histone deacetylase inhibitor (HDACI) activity; its major metabolite 2-pyrrolidinone-n-butyric acid (PBA) does, though to a lesser degree than valproic acid [40]. Lacosamide induces histone hyperacetylation *in vitro* though it is not a direct inhibitor of HDAC1 [41]. While HDACIs may have the potential to cause reactivation of chromosomally integrated HHV-6, it seems unlikely that our patient’s antiepileptic medications precipitated such a reactivation [42]. Conversation with the patient’s wife suggested that his behavioural problems had been ongoing for many years, even before the antiepileptics were started. Clinical improvement in this case did not correlate with the administration of antiviral medications, therefore his waxing and waning encephalopathy was thought to be due to his TBI and the sequelae of cerebrovascular disease rather than active HHV-6 encephalitis on a background of iciHHV-6.

## Conclusion

Human herpesvirus 6B is ubiquitous virus, which causes self-limited disease in childhood and rarely reactivates later in life in immunocompetent individuals. Approximately 1 % of the population carries chromosomally integrated HHV-6. Detection of HHV-6 DNA in the CSF of patients with iciHHV-6 is not diagnostic of encephalitis since routine PCR assays can detect integrated virus. This may lead to unnecessary treatment with potentially toxic antiviral medications if symptoms are equivocal and may be better explained by another condition. Clinicians should be cognizant of the possibility of iciHHV-6 and obtain a whole blood chromosomal integration assay in cases where high-grade DNAemia persists despite antiviral therapy. Ganciclovir resistance in HHV-6 is rare and foscarnet resistance has only been described *in vitro*. The decision to continue treatment is generally based on clinical judgement, as the presence of iciHHV-6 does not exclude HHV-6 encephalitis and mRNA-based testing is required to determine if the virus is actively replicating.

## References

[1] Zerr D, Meier A, Selke S, Frenkel L, Huang ML (2005). A population-based study of primary human herpesvirus 6 infection. N Engl J Med.

[2] Kotton CN, Hirsch MS, Jameson J, Fauci A, Kasper D, Hauser S, Longo D (2018). Harrison’s Principles of Internal Medicine, 20e.

[3] Agut H, Bonnafous P, Gautheret-Dejean A (2016). Human Herpesviruses 6A, 6B, and 7. Microbiol Spectr 4.

[4] Ablashi D, Agut H, Alvarez-Lafuente R, Clark DA, Dewhurst S (2014). Classification of HHV-6A and HHV-6B as distinct viruses. Arch Virol.

[5] Arbuckle JH, Medveczky MM, Luka J, Hadley SH, Luegmayr A (2010). The latent human herpesvirus-6A genome specifically integrates in telomeres of human chromosomes *in vivo* and *in vitro*. Proc Natl Acad Sci U S A.

[6] Pellett P, Ablashi D, Ambros P, Agut H, Caserta M (2012). Chromosomally integrated human herpesvirus 6: Questions and answers. Rev Med Virol.

[7] Lee SO, Brown RA, Razonable R (2012). Chromosomally integrated human herpesvirus-6 in transplant recipients. Transpl Infect Dis.

[8] Pruksananonda P, Hall C, Insel R, McIntyre K, Pellett P (1992). Primary Human Herpesvirus 6 Infection in Young Children. N Engl J Med.

[9] Aimola G, Beythien G, Aswad A, Kaufer BB (2020). Current understanding of human herpesvirus 6 (HHV-6) chromosomal integration. Antiviral Res.

[10] Epstein LG, Shinnar S, Hesdorffer DC, Nordli DR, Hamidullah A (2012). Human herpesvirus 6 and 7 in febrile status epilepticus: the FEBSTAT study. Epilepsia.

[11] Ward KN, Hill JA, Hubacek P, Camara R de la, Crocchiolo R (2019). Guidelines from the 2017 European Conference on Infections in *Leukaemia* for management of HHV-6 infection in patients with hematologic malignancies and after hematopoietic stem cell transplantation. Haematologica.

[12] Dougan C, Ormerod I (2004). A neurologist’s approach to the immunosuppressed patient. J Neurol Neurosurg Psychiatry.

[13] Razonablea RR, Zerrb DM (2009). HHV-6, HHV-7 and HHV-8 in solid organ transplant recipients. Am J Transplant.

[14] Lautenschlager I, Razonable R (2012). Human herpesvirus-6 infections in kidney, liver, lung, and heart transplantation: Review. Transpl Int.

[15] Kesely KR, Pantaleo A, Turrini FM, Olupot-Olupot P, Low PS (2016). Inhibition of an erythrocyte tyrosine kinase with imatinib prevents *Plasmodium falciparum egress* and *Terminates parasitemia*. PLoS One.

[16] Hall CB, Caserta MT, Schnabel K, Shelley LM, Marino AS (2008). Chromosomal integration of human herpesvirus 6 is the major mode of congenital human herpesvirus 6 infection. Pediatrics.

[17] Clark DA (2016). Clinical and laboratory features of human herpesvirus 6 chromosomal integration. Clin Microbiol Infect Off Publ Eur Soc Clin Microbiol Infect Dis.

[18] Luppi M, Marasca R, Barozzi P, Ferrari S, Ceccherini-Nelli L (1993). Three cases of human herpesvirus-6 latent infection: Integration of viral genome in peripheral blood mononuclear cell DNA. J Med Virol.

[19] Endo A, Watanabe K, Ohye T, Suzuki K, Matsubara T (2014). Molecular and virological evidence of viral activation from chromosomally integrated human herpesvirus 6A in a patient with X-linked severe combined immunodeficiency. Clin Infect Dis an Off Publ Infect Dis Soc Am.

[20] Ohye T, Kawamura Y, Inagaki H, Yoshikawa A, Ihira M (2016). A simple cytogenetic method to detect chromosomally integrated human herpesvirus-6. J Virol Methods.

[21] Miura H, Kawamura Y, Kudo K, Ihira M, Ohye T (2015). Virological analysis of inherited chromosomally integrated human herpesvirus-6 in three hematopoietic stem cell transplant patients. Transpl Infect Dis.

[22] Torelli G, Barozzi P, Marasca R, Cocconcelli P, Merelli E (1995). Targeted integration of human herpesvirus 6 in the p arm of chromosome 17 of human peripheral blood mononuclear cells in vivo. J Med Virol.

[23] Clark DA, Nacheva EP, Leong HN, Brazma D, Li YT (2006). Transmission of integrated human herpesvirus 6 through stem cell transplantation: implications for laboratory diagnosis. J Infect Dis.

[24] Strenger V, Caselli E, Lautenschlager I, Schwinger W, Aberle SW (2014). Detection of HHV-6-specific mRNA and antigens in PBMCs of individuals with chromosomally integrated HHV-6 (ciHHV-6. Clin Microbiol Infect Off Publ Eur Soc Clin Microbiol Infect Dis.

[25] Mori T, Tanaka-Taya K, Satoh H, Aisa Y, Yamazaki R (2009). Transmission of chromosomally integrated human herpesvirsus 6 (HHV-6) variant A from a parent to children leading to misdiagnosis of active HHV-6 infection. Transpl Infect Dis.

[26] Hill JA, Ikoma M, Zerr DM, Basom RS, Peddu V (2019). RNA sequencing of the in vivo human Herpesvirus 6B transcriptome to identify targets for clinical assays distinguishing between latent and active infections. J Virol.

[27] Ward K, Leong HN, Nacheva E, Howard J, Atkinson C (2006). Human Herpesvirus 6 chromosomal integration in immunocompetent patients results in high levels of viral DNA in blood, SERA, and hair follicles. J Clin Microbiol.

[28] Ljungman P (2017). Chromosomally integrated HHV-6: a new piece of the puzzle. Blood.

[29] Leber AL, Everhart K, Balada-Llasat JM, Cullison J, Daly J (2016). Multicenter evaluation of Biofire Filmarray Meningitis/encephalitis panel for detection of bacteria, viruses, and yeast in cerebrospinal fluid specimens. J Clin Microbiol.

[30] Green DA, Pereira M, Miko B, Radmard S, Whittier S (2018). Clinical significance of human herpesvirus 6 positivity on the Filmarray meningitis/encephalitis panel. Clin Infect Dis.

[31] Takano K, Ogata M, Kawano R, Satou T, Nashimoto Y (2018). Comparison of HHV-6 DNA detection in plasma and whole blood in allogeneic hematopoietic stem cell transplant recipients: frequent false-positive results for active HHV-6 infection using whole blood samples. Int J Hematol.

[32] Greninger AL, Naccache SN, Pannaraj P, Jerome KR, Dien Bard J (2019). The brief case: inherited chromosomally integrated human herpesvirus 6 (HHV-6) in the age of multiplex HHV-6 testing. J Clin Microbiol.

[33] Zerr DM, Fann JR, Breiger D, Boeckh M, Adler AL (2011). HHV-6 reactivation and its effect on delirium and cognitive functioning in hematopoietic cell transplantation recipients. Blood.

[34] Prichard MN, Whitley RJ (2014). The development of new therapies for human herpesvirus 6. Current Opinion in Virology.

[35] Komatsu TE, Pikis A, Naeger LK, Harrington PR (2014). Resistance of human cytomegalovirus to ganciclovir/valganciclovir: a comprehensive review of putative resistance pathways. Antiviral Res.

[36] Lurain NS, Chou S (2010). Antiviral drug resistance of human cytomegalovirus. Clin Microbiol Rev.

[37] Hiramatsu H, Suzuki R, Yamada S, Ihira M, Isegawa Y (2015). Analysis of ganciclovir-resistant human herpesvirus 6B clinical isolates using quenching probe PCR methodology. Antimicrob Agents Chemother.

[38] Rodriguez J, Casper K, Smallwood G, Stieber A, Fasola C (2007). Resistance to combined ganciclovir and foscarnet therapy in a liver transplant recipient with possible dual-strain cytomegalovirus coinfection. Liver Transpl.

[39] Bonnafous P, Naesens L, Petrella S, Gautheret-Dejean A, Boutolleau D (2007). Different mutations in the HHV-6 DNA polymerase gene accounting for resistance to foscarnet. Antivir Ther.

[40] Eyal S, Yagen B, Sobol E, Altschuler Y, Shmuel M (2004). The activity of antiepileptic drugs as histone deacetylase inhibitors. Epilepsia.

[41] Granit A, Tetro N, Shmuel M, Peretz T, Eyal S (2018). Lacosamide at therapeutic concentrations induces histone hyperacetylation *in vitro*. Epilepsia open.

[42] Politikos I, McMasters M, Bryke C, Avigan D, Boussiotis VA (2018). Possible reactivation of chromosomally integrated human herpesvirus 6 after treatment with histone deacetylase inhibitor. Blood Adv.

